# Host–Parasite Association Dynamics Influence Dispersal and Population Genetics of Little Brown Myotis (*Myotis lucifugus*, Le Conte 1831) Ectoparasites

**DOI:** 10.1002/ece3.71233

**Published:** 2025-04-27

**Authors:** Alexandra H. Sauk, Hugh G. Broders

**Affiliations:** ^1^ Department of Biology University of Waterloo Waterloo Ontario Canada

**Keywords:** biogeography, Chiroptera, Ischnopsyllidae, Spinturnicidae, transmission

## Abstract

Host–parasite relationships can affect the dispersal and transmission of parasites. *Myodopsylla insignis* (Rothchild, 1903), a bat flea, and *Spinturnix americanus* (Banks, 1902), a bat wing mite, are two common ectoparasites of the little brown myotis (*Myotis lucifugus,* Le Conte 1831) that differ in life cycles and time spent on the host. Our goal was to compare how life history traits and host–parasite relationships influence the genetic structure and biogeography of co‐infecting ectoparasites using *S. americanus* mites and *M. insignis* fleas that feed on *Myotis lucifugus* bats. Ectoparasites were collected from bats captured at maternity roosts between 2010 and 2017 in Atlantic Canada and sequenced for the cytochrome oxidase c subunit 1 gene. We barcoded 223 *S. americanus* and 87 *M. insignis* specimens and examined their genetic diversity, genetic structure, and biogeography. We found evidence of a weak association between geographic distance and sequence divergence between Labrador and Nova Scotia for *M. insignis* and evidence of regional differentiation between the island of Newfoundland and the mainland for *S. americanus*, similar to previous findings for *M. lucifugus* . In terms of biogeography, *M. insignis* likely underwent historical population expansion, particularly in Labrador, whereas *S. americanus* may have undergone historical population expansion or selection. Our study highlights how host–parasite relationships are influenced at multiple scales by both host and parasite biology and how an understanding of both host and parasite informs predictions on how these dynamics will be affected by disturbances.

## Graphical abstract

*Spinturnix americanus* and *Myodopsylla insignis* are two common ectoparasites of little brown myotis ( *Myotis lucifugus* ) with different life history strategies. These ectoparasites exhibit corresponding differences in genetic diversity and dispersal with their hosts across Atlantic Canada.

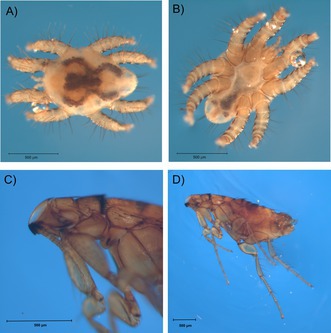

## 1 Introduction

Each species in a host–parasite relationship has unique traits that shape the relationship dynamics and the degree of congruence of their genetic variation (Mazé‐Guilmo et al. 2016). Parasite population genetic structure is generally considered to be dependent on host dispersal (Mazé‐Guilmo et al. 2016), but several other factors influence the patterns of genetic variation between host and parasite. Parasite traits that affect their genetic structure include population size, dispersal ability, life cycle complexity (e.g., number of intermediate or reservoir hosts), reproductive mode (e.g., self‐fertilizing or sexually reproducing), and their susceptibility to stochastic events (Nadler 1995; Engelbrecht et al. 2016; Cole and Viney 2019). The abundance, distribution, vagility, and movement behavior of the host may also affect the genetic structure of parasites through influences on dispersal and population connectivity (Nadler 1995; McCoy et al. 2003; Criscione et al. 2005).

The ectoparasites of little brown myotis ( *M. lucifugus* ) provide an opportunity to investigate how differences in parasite life history and host dependence affect parasite biogeographic patterns and genetic structure. Little brown myotis are small, insectivorous bats with a wide geographic range in North America (Davis and Hitchcock 1959; Fenton and Barclay 1980). Female bats form maternity groups during the summer months, and males roost individually or in small groups (Fenton and Barclay 1980; Anthony et al. 1981; Broders and Forbes 2004). Males and females of the species congregate during autumn at swarming sites, which is followed by overwinter hibernation (Fenton and Barclay 1980; Burns and Broders 2015a, 2015b). Previous studies of the population genetic structure of *M. lucifugus* have shown low to moderate levels of population differentiation because of the nature of their aggregations (Burns et al. 2014; Johnson et al. 2015; McLeod et al. 2015; Vonhof et al. 2015). Within Atlantic Canada, little brown myotis inhabit both mainland and island locations during the summer, making use of both natural and anthropogenic structures for roosts. Previous work by McLeod et al. (2015) identified that the oceanic straits between the mainland and islands of the Gulf of the St. Lawrence are not an impenetrable barrier to gene flow for *M. lucifugus;* however, the bats on the island of Newfoundland exhibited less genetic diversity and less population genetic connectivity to bats from elsewhere. Regional genetic differentiation between mainland and island groups may be more evident in bat parasites due to their shorter generation time and more limited dispersal (Nadler 1995; McCoy et al. 2005; Nieberding and Olivieri 2007).

During the last glacial maximum of the Pleistocene, refugia extended along the eastern coast of North America from just south of Nova Scotia that could have provided suitable forest habitat for bats (Shaw et al. 2006; Burns et al. 2014). The high vagility of bats would have allowed them to track the recolonizations of forests along this eastern coast as the ice receded (Burns et al. 2014). Evidence supports the contention that this recolonization from refugia led to a subsequent population expansion in eastern Canada (Burns et al. 2014). A subset of early migrants to the region would likely have been infected with parasites, creating founding populations and the potential for coinciding parasite population expansions.

**FIGURE 1:**
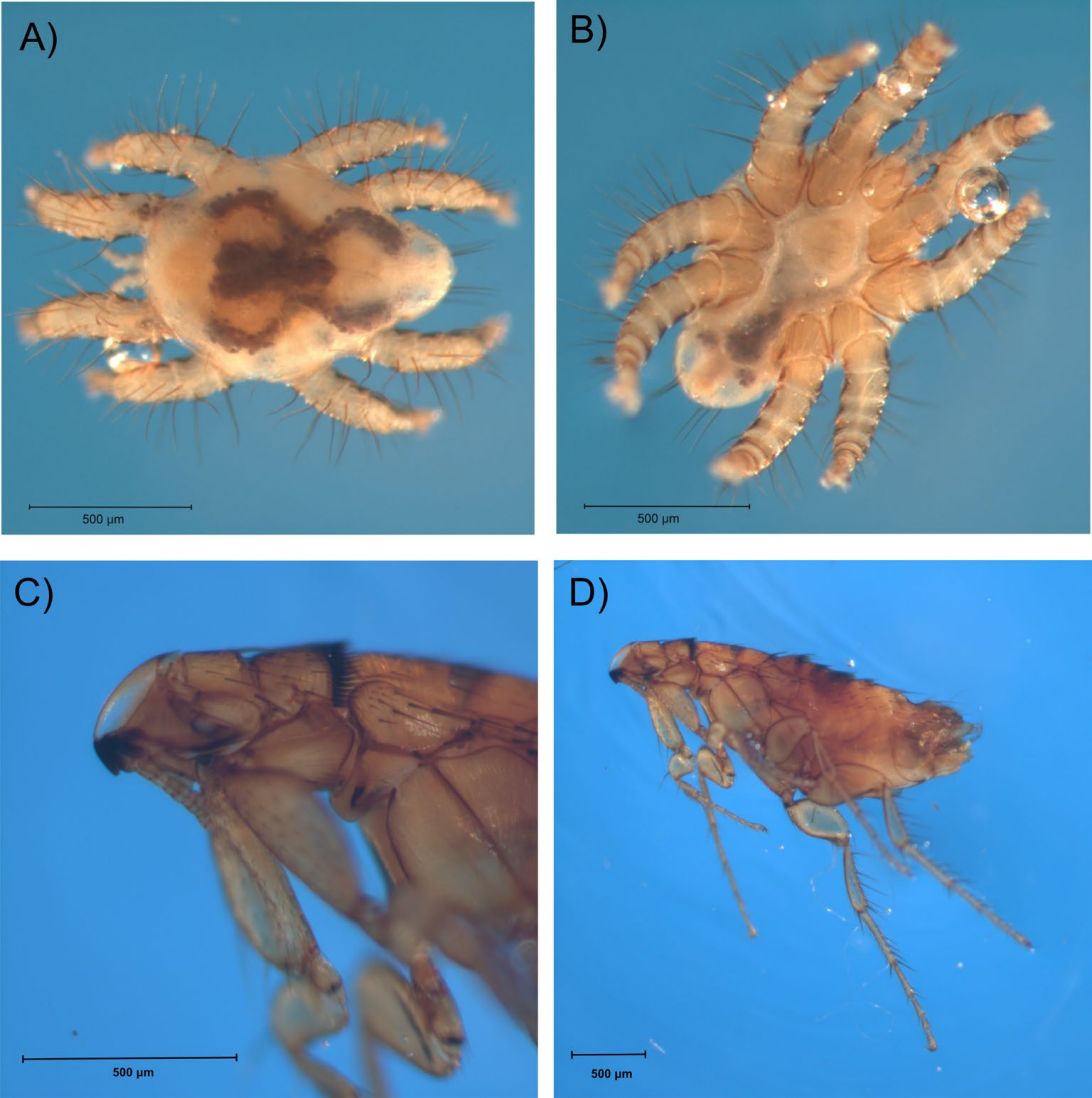
Representative specimen photos of *Spinturnix americanus* (A) dorsal, (B) ventral; and *Myodopsylla insignis* (C) head region, and (D) side view.

*Spinturnix americanus* is a common ectoparasitic mite (Figure 1A,B) found on the wings of bats in North America, having been recorded from *Antrozous pallidus* (Whitaker and Easterla 1975), *Corynorhinus mexicanus* (Villegas‐Guzman et al. 2005), and several *Myotis* species (Ubelaker 1966; Whitaker 1973; Czenze and Broders 2011) among others. It has been found infecting *M. lucifugus* from central to eastern Canada (Poissant and Broders 2008; Czenze and Broders 2011; Webber, Czenze, et al. 2015), with increased infection on adult female bats and juveniles of both sexes (Czenze and Broders 2011). Spinturnicidae mites are permanent ectoparasites completing their life cycle attached to the membranes of their host (Rudnik 1960; Dowling 2015), often with high host specificity at the species or genus level (Christe et al. 2000; Ter Hofstede and Fenton 2005; Bruyndonckx, Dubey, et al. 2009). The mites are transmitted between bat hosts vertically from mother to offspring or horizontally during physical contact between bats, such as during roosting aggregations or copulation (Christe et al. 2000; Ter Hofstede and Fenton 2005). The genetic population structure of spinturnids appears to be dependent on the movement and social behavior of their host species (Bruyndonckx, Dubey, et al. 2009; Van Schaik et al. 2014, 2015; Zamora‐Mejías et al. 2022). The reduced contact between roost groups of *Myotis bechsteini* (Bechstein's bat) corresponds to higher genetic structuring in *Spinturnix bechsteini* (Bruyndonckx, Henry, et al. 2009), whereas the movement of migratory females of *Leptonycteris yerbabuenae* (lesser long‐nosed bat) allows for gene flow in *Periglischrus paracaligus* (Zamora‐Mejías et al. 2022). Due to the dependence of *Spinturnix americanus* on their host, we would expect their genetic structure to resemble the low genetic structure of *M. lucifugus* in Atlantic Canada.

*Myodopsylla insignis* fleas (Figure 1C,D) are found infecting bats in North America, including *M. lucifugus* (Dick et al. 2003; Poissant and Broders 2008; Eckerlin and Gardner 2021), *M. septentrionalis* (Chilton et al. 2000; Czenze and Broders 2011; Gikas et al. 2011) and *Eptesicus fuscus* (Phillips 1966; Dick et al. 2003). These fleas are parasitic as adults, with early development of larvae and pupae occurring in the roost substrate (Smith and Clay 1988). Once development is complete and adult *M. insignis* emerge from their pupal state, they must locate a bat host (Smith and Clay 1988). Adult *M. insignis* are commonly found on *M. lucifugus* after the bats have left the roost for foraging or swarming (Poissant and Broders 2008; Czenze and Broders 2011; Webber, McGuire, et al. 2015). This may allow for dispersal between roosts; however, bats are also known to switch roosts to avoid heavy infestations of ectoparasites and their associated energetic costs (Wilkinson 1985; Lewis 1995; Bartonička 2008). If fleas are prevented from dispersing with their hosts, coupled with flea development occurring off the host, their susceptibility to stochastic events and genetic drift may increase, affecting their genetic structure (Nadler 1995; Mazé‐Guilmo et al. 2016). While *M. insignis* is associated with multiple bat species in North America (Phillips 1966; Smith and Clay 1988; Chilton et al. 2000; Poissant and Broders 2008; Czenze and Broders 2011), no studies have yet determined the genetic structure for this flea species, limiting insight into if and how dispersal and gene flow occurs between roosts.

By comparing the genetic diversity of two co‐infecting, and taxonomically distinct ectoparasites, we can observe the influence of their different reproductive strategies. Both ectoparasites are expected to synchronize their reproduction with that of their hosts (Marshall 1982; Smith and Clay 1988; Christe et al. 2000; Orlova et al. 2018) but exhibit different modes of reproduction. *Myodopsylla insignis* is oviparous, with females likely producing and laying many eggs a day and potentially hundreds in a lifetime, similar to other flea species (Marshall 1982). Conversely, *Spinturnix americanus*, like other spinturnid mites, is viviparous, where female spinturnid mites internally embryonate eggs and larvae before giving birth to the blood‐feeding protonymph (Orlova et al. 2018). We expect these differences in life history strategies will be reflected in the genetic diversity of these ectoparasites.

The goal of this study was to compare how life history traits and host–parasite relationships influence the genetic structure and biogeography of co‐infecting ectoparasites. Our aim was to characterize the genetic structure present in *S. americanus* and *M. insignis* in relation to the genetic structure of the host, *M. lucifugus* , and assess their regional biogeographic patterns. Given the permanent nature of the host–parasite relationship between *S. americanus* and *M. lucifugus* , we predicted that *S. americanus* would have similarly low levels of genetic differentiation to *M. lucifugus* . Conversely, we predicted *M. insignis* would show at least somewhat higher levels of genetic structure as portions of its life cycle are spent in the roost, which limits transmission to adult fleas. *Myotis lucifugus* experienced a population expansion approximately 12,500 to 1250 years before the present after moving into the region after the last deglaciation, and we hypothesized that there would be evidence that *M. insignis* and *S. americanus* had experienced similar population expansions and predicted there would be genetic signatures of this expansion indicated by deviations from neutral theory and characteristic starburst haplotype distributions where high‐frequency haplotypes are central to several low‐frequency haplotypes. Understanding the host–parasite dynamics of these species provides insight into the factors affecting parasite genetics at different geographic scales and provides an important conservation context for the movement patterns of an elusive and endangered host.

## 2 Materials and Methods

### 2.1 Specimen Collection

*Spinturnix americanus* and *Myodopsylla insignis* specimens were collected from *Myotis lucifugus* bats in Atlantic Canada between 2010 and 2017 at maternity roosts as part of bat population research projects (for examples, see Poissant and Broders 2008; Poissant et al. 2010; Czenze and Broders 2011; Burns and Broders 2014, 2015a, 2015b; Burns et al. 2014; McLeod et al. 2015; Sunga et al. 2024). Bats were caught in either mist nets (Avinet, Dryden, New York, USA) or harp traps (Austbat Research Equipment, Lower Plenty, VIC, Australia) positioned close to maternity roosts. Ectoparasites were sampled from the wings, ears, and body fur of the bats during visual inspections of the bats as part of standard procedures to measure bat mass and forearm length. Collected ectoparasites were stored in 70% ethanol in the field and stored at −80°C for long‐term storage. Identification of specimens was confirmed using microscopy and diagnostic characteristics (Whitaker 1982). *Spinturnix americanus* was distinguished from other mites found on bats in Atlantic Canada by the four pairs of legs that were large in relation to their body size and organized radially around the idiosoma and the presence of tiny ventral setae and proximal dorsal setae of femora I and II (Rudnik 1960; Whitaker 1982). *Myodopsylla insignis* was identified based on the presence of two genal spines on the front portion of the head instead of the back, a truncate maxilla, and the presence of a pronotal comb (Whitaker 1982).

### 2.2 Specimen Sampling

**FIGURE 2:**
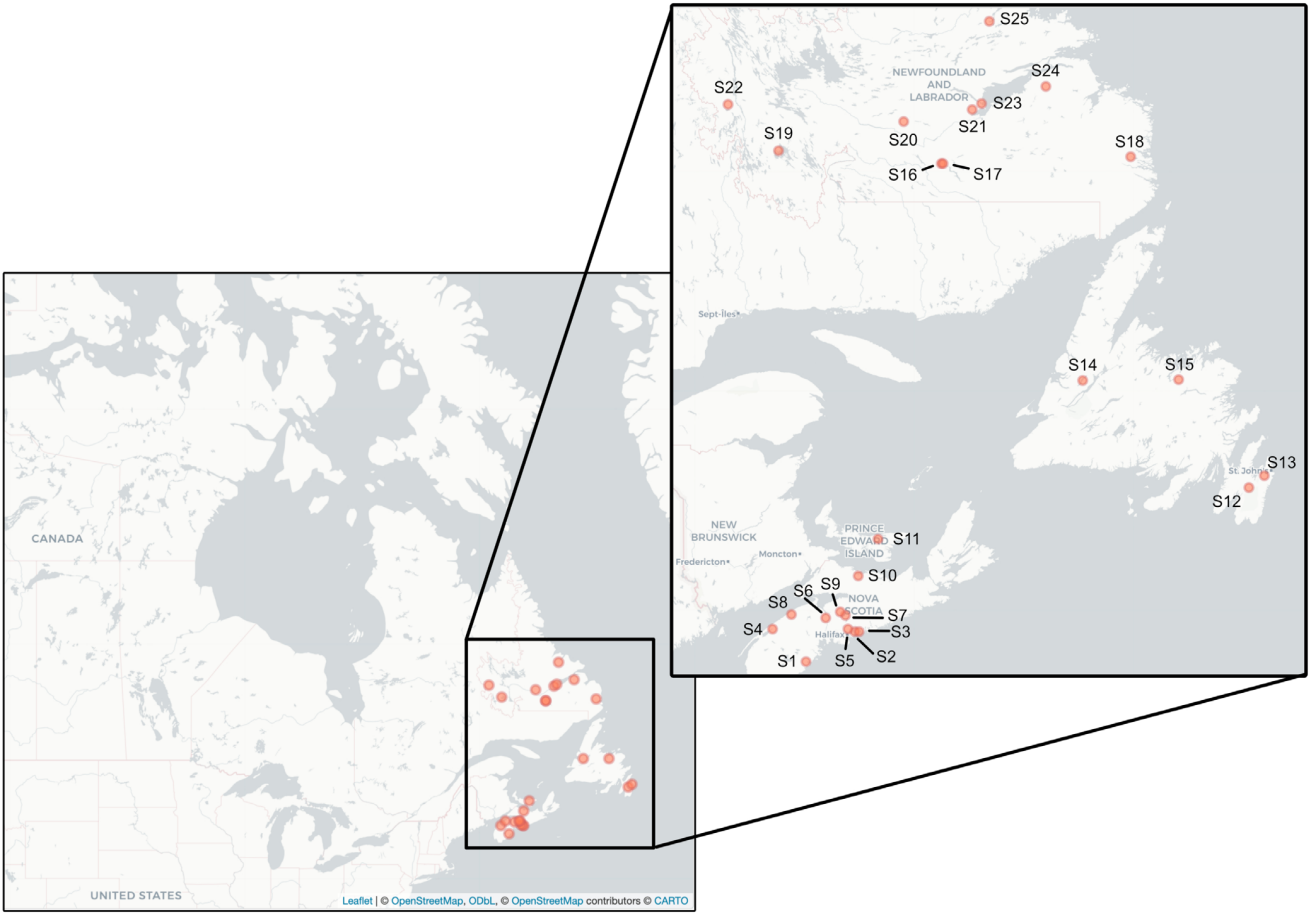
Study area map with 25 collection sites across Atlantic Canada for *M. insignis* and *S. americanus* between 2010 and 2017. Ectoparasite samples were collected from ten sites in Nova Scotia (S1–Mill Village, S2–Mineville, S3–West Chezzetcook, S4–Annapolis Royal, S5–Waverley, S6–Martock, S7–Renfrew, S8–Vault Cave, S9–Rawdon, S10–Tatamagouche), one site in Prince Edward Island (S11–Head of Hillsborough), four sites on the island of Newfoundland (S12–Salmonier Nature Park, S13–Paddy's Pond, S14–Pynns Brook, S15–Notre Dame Provincial Park), and ten sites from the mainland of Labrador (S16–Anne Marie Lake, S17–Minipi Lake, S18–Charlottetown Junction, S19–Wabush, S20–Cache River, S21–Birch Brook, S22–Grand Lake Rd., S23–Northwest River, S24–English River, S25–Kaipokok River). Number of ectoparasite samples from each site is available in Tables S1 and S2. The map was visualized using the package *leaflet* in R with map tiles by CARTO (CC BY 3.0) and map data from OpenStreetMap (openstreetmap.org/copyright).

Specimens for this study were restricted to those collected from maternity roosts where female adult *M. lucifugus* give birth and raise their young (Davis and Hitchcock 1959; Fenton and Barclay 1980). *Spinturnix americanus* specimens were included from nine sites in Nova Scotia, one site in Prince Edward Island, 10 sites in Labrador, and four sites from the island of Newfoundland. *Myodopsylla insignis* specimens were included from five sites in Nova Scotia, five sites in Labrador, and three sites from the island of Newfoundland. Collection locations were mapped using the *leaflet* R package (Figure 2; R Core Team 2023; Cheng et al. 2024).

*Spinturnix americanus* specimens were selected from different hosts (i.e., individual *M. lucifugus* ) where possible to sample a greater range of specimens. However, in some locations where fewer than five (< 5) hosts were sampled, multiple mites were sequenced from certain hosts (Bruyndonckx, Henry, et al. 2009; van Schaik et al. 2011). All *M. insignis* were collected from hosts, and multiple fleas were sequenced from some hosts; however, early flea development occurs in the roost, so this is not anticipated to affect sample diversity (Marshall 1982; Smith and Clay 1988). A maximum of four mites or five fleas were sequenced from any individual bat, with 57.9% of mite specimens and 50.5% of flea specimens being the sole mite or flea sequenced from the host. For 15 of 195 host bats, both mites and fleas were sequenced. Only locations with four or more specimens of either *S. americanus* or *M. insignis* were included, as minimum sample sizes of three were needed to calculate Tajima's *D* and test for deviations from neutrality (Tajima 1989). The number of specimens successfully sequenced from each site is included in Tables S1 and S2.

### 2.3 Cytochrome c Oxidase Subunit I (COI) Gene Sequencing

*Spinturnix americanus* and *M. insignis* specimens selected for sequencing were prepared as per Canadian Centre for DNA Barcoding (CCDB) guidelines and submitted to the CCDB for COI DNA barcoding. DNA extraction, polymerase chain reaction (PCR) amplification, and Sanger DNA sequencing were completed by the CCDB. DNA extractions were carried out using the protocol outlined in Ivanova et al. (2006) with adjustments for extraction of arthropod DNA (Ivanova and Hebert 2006; Ivanova et al. 2006). PCR amplification was conducted using the protocol by Ivanova and Grainger (2007). Forward and reverse COI mitochondrial DNA (mtDNA) gene primers used for both *M. insignis* and *S. americanus* were C_LepFolF 5'‐ATTCAACCAATCATAAAGATATTGG‐3' and 5'‐TAAACTTCTGGATGTCCAAAAAATCA‐3' (Hernández‐Triana et al. 2014). Specimen metadata was deposited into the Barcode of Life Database (BOLD) (Ratnasingham and Hebert 2007). Sequences greater than 600 bp were aligned using the default settings for MUSCLE (Edgar 2004) in MEGA X (Kumar et al. 2018).

### 2.4 *Myotis lucifugus* Sequences

Sequences of the hypervariable region II (HVII) of the mtDNA control region from previous work by McLeod et al. (2015) were used to compare the population genetic structure of the mites and fleas to their host, *M. lucifugus* . Tissue samples from *M. lucifugus* were collected between 2008 and 2012 from 21 sites in the provinces of Quebec, Nova Scotia, Prince Edward Island, and Newfoundland and Labrador (McLeod et al. 2015). Collection of bat samples and ectoparasites overlapped in some years, but ectoparasites were not selected based on whether they were collected from bats with sequences from McLeod et al. (2015). Haplotype sequences were downloaded from NCBI GenBank, and a dataset was created based on the number of haplotypes present at each site (McLeod et al. 2015). Sequences were aligned using default settings for MUSCLE (Edgar 2004) in MEGA X (Kumar et al. 2018) with gaps removed from sites 16 and 22.

### 2.5 Genetic Diversity

Genetic diversity statistics were calculated for the ectoparasites and *M. lucifugus*. Mean and maximum pairwise differences between sequences for *S. americanus* , *M. insignis*, and *M. lucifugus* were calculated using MEGA X (Kumar et al. 2018) and the Tamura–Nei distance model (Tamura and Nei 1993). Nucleotide diversity, which is the average number of nucleotide differences per site between two sequences (Nei and Li 1979), and haplotype diversity, the probability that two randomly sampled alleles will be different (de Jong et al. 2011), were calculated using DnaSP (version 6.12.03) (Librado and Rozas 2009).

### 2.6 Genetic Structure

To evaluate whether spatial processes contribute to genetic structure for *M. insignis* and *S. americanus* , the correlation between geographic distance and sequence divergence was calculated using the geo‐distance correlation tool on BOLD using the Kimura 2‐Parameter distance model (Kimura 1980). The geo‐distance tool aligns sequences using the BOLD aligner, which is an amino acid‐based Hidden Markov Model (HMM). The geo‐distance correlation tool conducts a Mantel Test (Mantel 1967) between the geographic distance matrix (in km, from collection site latitude and longitude) and the genetic divergence matrix, and a comparison of spread using the minimum spanning tree of collection sites and the maximum intraspecific divergence (Blagoev et al. 2016).

DnaSP (version 6.12.03) was used to format haplotype data for analysis in Arlequin. Maternity sites were used to assign sequences to populations, and regions were used for the group‐level structure. Three regions were included for *M. insignis* (Nova Scotia, Newfoundland, and Labrador) and four regions for *S. americanus* (Nova Scotia, Newfoundland, Labrador, and Prince Edward Island). Five regions were included for *M. lucifugus* based on capture locations in McLeod et al. (2015): Quebec, Nova Scotia, Newfoundland, Labrador, and Prince Edward Island.

To test for genetic differentiation between mainland regions and islands, Arlequin (version 3.5.2.2) was used to calculate the analysis of molecular variance (AMOVA) (Excoffier and Lischer 2010). AMOVA calculates the proportion of variance among groups (Φ_CT_), among populations within groups (Φ_SC_), and within populations (Φ_ST_) and evaluates the statistical significance by randomly permutating the sequences among populations (Der Tzeng et al. 2004). At low numbers of populations per group, it can be difficult to detect between‐group structure with analysis of molecular variance due to statistical limitations with the possible number of permutations of sampled populations (Fitzpatrick 2009). For this study, all mites or fleas collected at a maternity roost were considered a population (referred to as a roost or roost site) and the regions were considered groups. A power analysis was conducted as per Fitzpatrick (2009) to ensure that the number of maternity roosts within regions would provide a sufficient number of permutations to result in *p*‐values of < 0.05 if a higher‐level population structure was present. Arlequin (version 3.5.2.2) was also used to calculate pairwise differentiation (*F*_ST_) values for *M. insignis* and *S. americanus* as a measure of genetic differentiation between roosts.

### 2.7 Demographic History

To determine whether there was evidence that ectoparasites underwent a historical population expansion, we inferred haplotype networks from the mtDNA sequences and conducted tests of neutrality. Maximum parsimony haplotype networks were constructed using the ‘haploNet’ function from the R package *pegas* using the haplotype frequencies to visualize the regional distribution of haplotypes (Paradis 2010). The ‘rmst’ function in the R package *pegas* was used to infer randomized minimum spanning trees (RMST) of the haplotypes and these trees were examined for starburst patterns that would indicate past range expansions (Paradis 2010, 2018; R Core Team 2023). The RMST method is an alternative to the minimum spanning network method for inferring haplotype networks that involve fewer alternative links, and resample the distance matrix with randomized reordering to remove ambiguity or bias that could be introduced by the order of the data (Paradis 2018). Haplotype networks were examined for starburst patterns, where a common central haplotype has many haplotypes branching from it that differ by one or two nucleotides. Starburst patterns are suggestive of demographic expansion (Yuan et al. 2010; Pulgarín‐R and Burg 2012). Tests of neutrality were conducted to determine whether the populations of *S. americanus* and *M. insignis* in Atlantic Canada are evolving randomly or are experiencing selective pressure or demographic changes. Tajima's *D* was calculated in Arlequin (version 3.5.2.2) for each population with four or more sequenced specimens. Tajima's *D*, Fu's *F*_S_, and Fu and L's D* and F* were calculated for all specimens sequenced from Atlantic Canada using DnaSP (version 6.12.03).

### 2.8 Ethics Statement

All bats were captured and released according to animal care protocols approved by the animal care committees of Saint Mary's University, Halifax, Nova Scotia, and the University of Waterloo, Waterloo, Ontario. Wildlife scientific research permits were obtained from the government agencies of the provinces of Nova Scotia, New Brunswick, Prince Edward Island, and Newfoundland and Labrador.

## 3 Results

### 3.1 Genetic Diversity

Cytochrome c oxidase subunit I (COI) mitochondrial DNA (mtDNA) gene alignment sequences of 653 and 611 nucleotides were generated for 87 *M. insignis* and 223 *S. americanus*, respectively, across all roosts. Genetic diversity was found to be greater for *M. insignis* than for *S. americanus* across all metrics. The COI sequences for *M. insignis* included 7.8% variable sites (51) whereas the sequences for *S. americanus* included 4.1% variable sites (25). There were 45 and 27 haplotypes identified for *M. insignis* and *S. americanus*, respectively. The nucleotide diversity or average pairwise difference between sequences for *M. insignis* was 0.006, with a maximum of 0.022 (Table 1). For *S. americanus* , the average pairwise difference between sequences was 0.004, with a maximum of 0.012. In comparison, the HVII sequences for *M. lucifugus* produced an alignment of 293 nucleotides with 51 variable sites. *Myotis lucifugus* exhibited a comparable haplotype diversity of 0.927. The pairwise difference between sequences was greater in *M. lucifugus* (mean = 0.02, max = 0.052); however, this may be attributable to a greater sample size (625 individuals) allowing for more variation to be captured and to the different gene regions.

**TABLE 1:** Summary statistics for sequence and haplotype diversity for *Spinturnix americanus*, and *Myodopsylla insignis* collected from *Myotis lucifugus* at maternity sites in Atlantic Canada between 2010 and 2017 and *Myotis lucifugus* sampled by McLeod et al. (2015).

Summary statistics	*Myodopsylla insignis*	*Spinturnix americanus*	*Myotis lucifugus*
No. of sequences	87	223	625
No. of variable sites	51	25	57
No. of haplotypes	45	27	109
Haplotype diversity	0.940	0.833	0.927
Average no. of nucleotide differences	4.001	2.406	5.573
Mean pairwise distance	0.006	0.004	0.020
Maximum pairwise distance	0.022	0.012	0.052

### 3.2 Genetic Structure

The distance between roosts ranged from 2 km (Anne Marie Lake, Labrador from 2011 and Minipi Lake, Labrador from 2012) to 1227 km (Mill Village, Nova Scotia, and Kaipokok River, Labrador). The Mantel test indicated a weak positive association between geographic distance and sequence divergence for *M. insignis* (*r* = 0.105, *p* = 0.01) whereas there was little to no association for *S. americanus* (*r* = 0.017, *p* = 0.01), suggesting fleas were more likely to have genetic structure among sites than mites.

Variation within roosts accounted for the greatest proportion of molecular variation for *M. insignis* and *S. americanus* (Tables 2 and 3). The Φ statistics were significantly different from zero (*p* < 0.01) for both species except for among roosts for *M. insignis* (Φ_SC_ = 0.011, *p* = 0.328). The low roost variation for *S. americanus* and *M. insignis* suggests gene flow among roosts. Some significant genetic differentiation is present for *M. insignis* and *S. americanus* between regions, 7.33% (*p* < 0.001) and 8.99% (*p* = 0.001), respectively, suggesting some regional differentiation in genetic structure and more restricted gene flow.

**TABLE 2:** . Analysis of molecular variance results for *Myodopsylla insignis* collected from *Myotis lucifugus* at 13 maternity sites in three regions (Nova Scotia, Newfoundland, and Labrador) within Atlantic Canada between 2010 and 2017.

Source of variation	Degrees of freedom	Sum of squares	Variance components	% Total variance	Φ‐statistics	*p*
Among regions	2	2.99	0.04	7.33	Φ_CT_ = 0.073	< 0.001
Among roosts	10	4.68	0.01	0.98	Φ_SC_ = 0.011	0.328
Within roosts	74	32.76	0.44	91.69	Φ_ST_ = 0.083	< 0.001

**TABLE 3:** . Analysis of molecular variance for *Spinturnix americanus* collected from *Myotis lucifugus* at 23 maternity sites in four regions (Nova Scotia, Prince Edward Island, Newfoundland, and Labrador) within Atlantic Canada between 2010 and 2017.

Source of variation	Degrees of freedom	Sum of squares	Variance components	Percentage of variation	Φ‐statistics	*p*
Among regions	3	8.43	0.04	8.99	Φ_CT_ = 0.010	0.001
Among roosts	20	14.74	0.04	10.19	Φ_SC_ = 0.112	< 0.001
Within roosts	199	69.35	0.35	80.82	Φ_ST_ = 0.192	< 0.001

For *M. lucifugus*, the greatest proportion of variation was also found within roosts (89.55%), followed by among roosts (6.60%), and among regions (3.85%, Table 4). The high within‐roost variance suggests that roost populations are well connected with gene flow between them and low inter‐roost differentiation. The genetic differentiation of *M. lucifugus* was significant at all hierarchical levels (*p* < 0.001).

**TABLE 4:** Analysis of molecular variance for *Myotis lucifugus* sequences of the hypervariable region II (HVII) of the mtDNA control region from sampled McLeod et al. (2015) from bats in Quebec, Nova Scotia, Prince Edward Island, Newfoundland, and Labrador.

Source of variation	Degrees of freedom	Sum of squares	Variance components	Percentage of variation	Φ‐statistics	*p*
Among regions	4	81.79	0.11	3.85	Φ_CT_ = 0.039	0.001
Among roosts	16	125.52	0.19	6.60	Φ_SC_ = 0.069	< 0.001
Within roosts	604	1532.45	2.54	89.55	Φ_ST_ = 0.105	< 0.001

Regional genetic differentiation is apparent for *S. americanus* between the island of Newfoundland and the mainland. Pairwise differentiation (*F*_ST_) was significant (*p* < 0.001) between Salmonier Nature Park and all roost sites in Labrador, seven of the nine roost sites in Nova Scotia, and one site in Prince Edward Island (Table S3). Pynn's Brook, Newfoundland, was significantly differentiated from seven of the eleven roost sites in Labrador but was not significantly differentiated from any site in Nova Scotia. Pairwise differentiation was significant for 22 roost site pairs between Nova Scotia and Labrador. These results suggest that *S. americanus* in Newfoundland experienced genetic differentiation from the mainland, with some differentiation occurring between Labrador and Nova Scotia as well.

There was less evidence of regional genetic differentiation for *M. insignis* , but results suggested that Anne Marie Lake and Charlottetown Junction, Labrador were significantly differentiated from roost sites in Nova Scotia (Table S4), and Anne Marie Lake, Labrador was differentiated from Salmonier Nature Park, Newfoundland (*p* < 0.001).

### 3.3 Demographic History

Tests of neutrality were conducted to determine if there was evidence that either ectoparasites experienced demographic expansion following the arrival of *M. lucifugus* in Atlantic Canada or if they were experiencing selection. Across all roost sites in Atlantic Canada, Tajima's *D* was significant for *M. insignis* (*D* = −2.056, *p* = 0.002), but not for *S. americanus* (*D* = −1.161, *p* > 0.10). Tajima's *D* was significant for two populations of *M. insignis*, Charlottetown Junction, Labrador (*D* = −1.658, *p* = 0.01) and Anne Marie Lake, Labrador (*D* = −2.129, *p* = 0.00), and approaching significance for the population from Rawdon, Nova Scotia (*D* = −1.464, *p* = 0.06). These negative Tajima's *D* results suggest that *M. insignis* may have undergone historical population expansion in these areas as a *D* < 0 can indicate a deviation from neutral theory, either as balancing selection or population growth (Tajima 1989). Tajima's *D* was nonsignificant for all other roost groups of *M. insignis* and all roost groups of *S. americanus*. Fu's *F*_S_ was significant for *M. insignis* (*F*_S_ = −42.25, *p* < 0.001) and *S. americanus* (*F*_S_ = −12.43, p 0.002). Fu and Li's D* and F* were significant for both *M. insignis* (D* = −3.926, *p* < 0.02; F* = −3.808, *p* < 0.02) and *S. americanus* (D* = −2.613, *p* < 0.05; F* = −2.379, *p* < 0.05). Negative values of Fu and Li's D* and F* indicate an excess of singleton haplotypes in the population which, similar to Tajima's *D*, suggests population expansion or selection (Fu and Li 1993), with the significant negative results for Fu's *F*_S_ supporting population expansion (Fu 1997). These results provide additional support for historical population expansion for *M. insignis* and suggest that it may have occurred for *S. americanus* as well.

**FIGURE 3:**
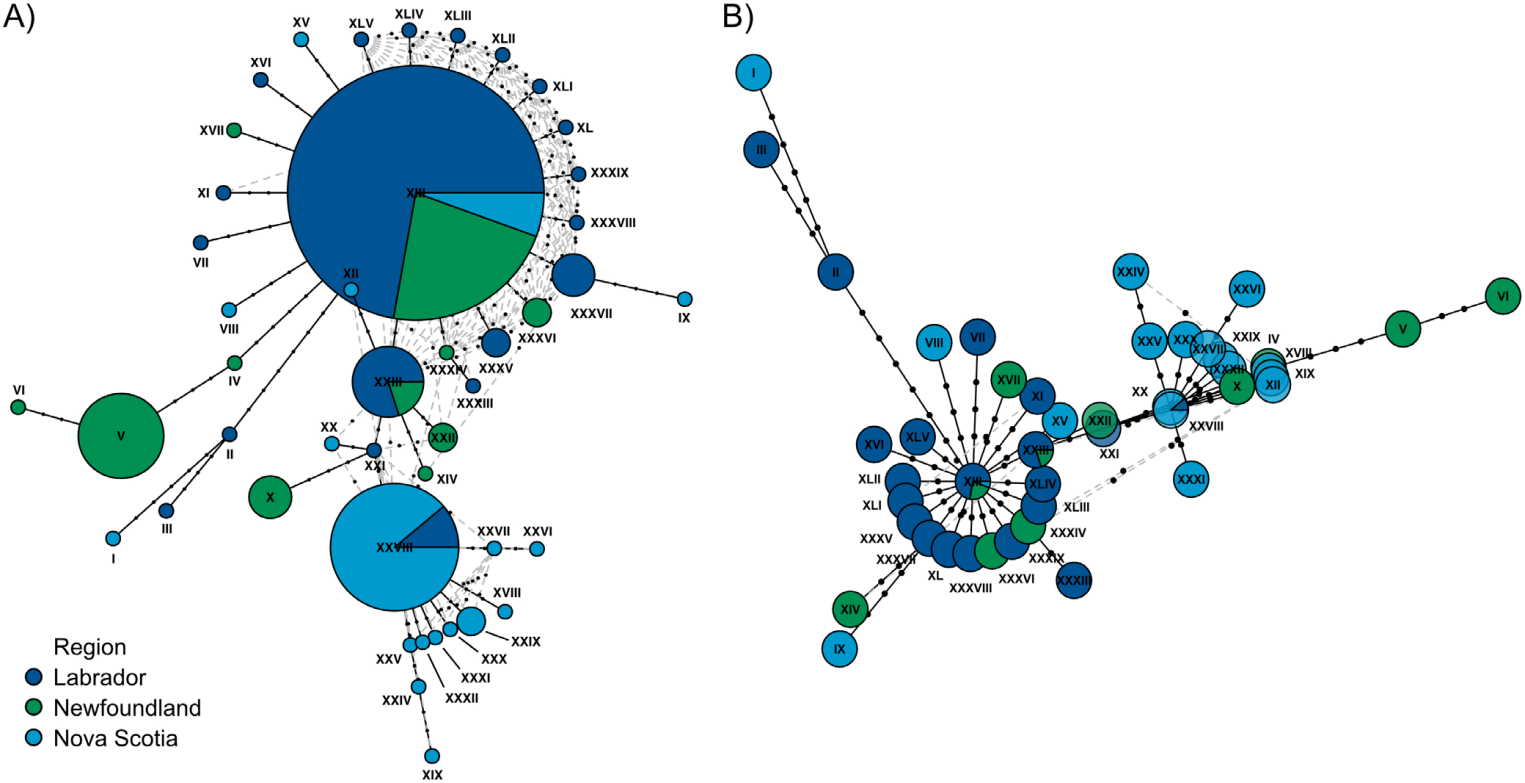
Visual representation of genetic relationships among *Myodopsylla insignis* individuals and geographic regions inferred using (A) maximum parsimony and (B) random minimized spanning tree algorithms. Individuals are color‐coded according to collection region and Roman numerals correspond to haplotype numbers. The size of circles in (A) corresponds to the frequency of each haplotype whereas circle sizes are standardized in (B) to allow for the visualization of the starburst patterns. Black dots on lines represent missing mutational steps between haplotypes with solid black lines representing primary links between haplotypes and gray dashed lines representing alternative links between haplotypes. The RMST algorithms produce fewer alternative links than the maximum parsimony method. Networks were calculated and visualized using *pegas* in RStudio (Paradis 2010; R Core Team 2023).

Of the 87 *M. insignis* sequenced, 40.2% were assigned singleton haplotypes, and 20.7% were assigned to the most abundant haplotype, XIII (Figure 3A). The haplotype networks for *M. insignis* exhibit starburst patterns around two haplotypes, XIII and XXVIII (Figure 3A,B), suggesting population expansion in Labrador and Nova Scotia. The haplotypes branching from haplotype XIII are primarily present in Labrador and Newfoundland, while the haplotypes branching from XXVIII are only present in Nova Scotia. The higher frequencies of XIII and XXVIII, coupled with the starburst patterns around these haplotypes, support the contention that these haplotypes are ancestral in Labrador and Nova Scotia, respectively.

**FIGURE 4:**
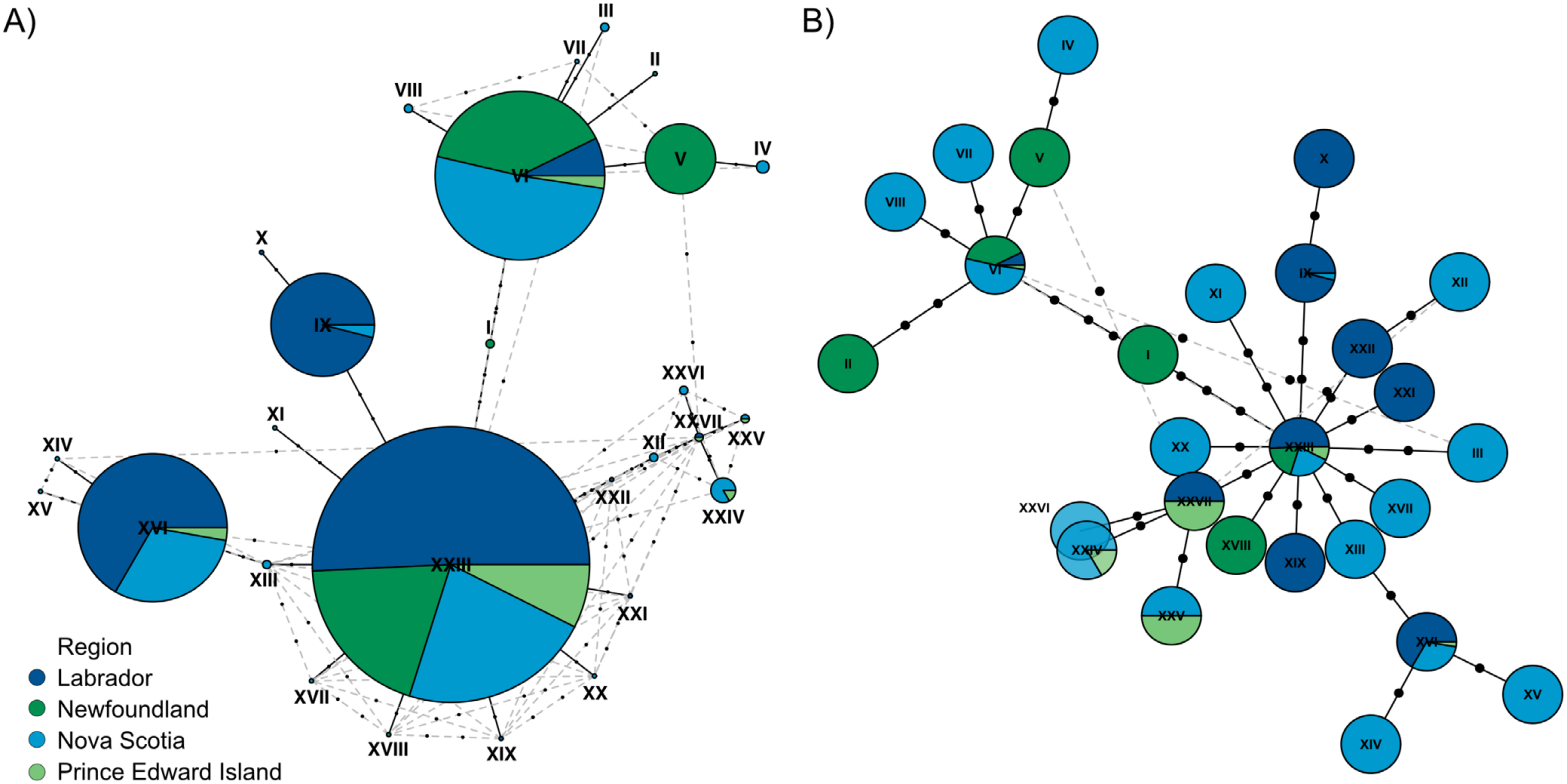
Visual representation of genetic relationships among *Spinturnix americanus* individuals and geographic regions inferred using (A) Maximum parsimony and (B) random minimized spanning tree algorithms. Individuals are color‐coded according to collection region and Roman numerals correspond to haplotype numbers. The size of circles in (A) corresponds to the frequency of each haplotype whereas circle sizes are standardized in (B) to allow for the visualization of the starburst patterns. Black dots on lines represent missing mutational steps between haplotypes with solid black lines representing primary links between haplotypes and gray dashed lines representing alternative links between haplotypes. The RMST algorithms produce fewer alternative links than the maximum parsimony method. Networks were calculated and visualized using *pegas* in RStudio (Paradis 2010, R Core Team 2023).

Of the 223 *S. americanus* sequenced, 83% were assigned one of the five most abundant haplotypes, with 30% assigned to haplotype XXIII alone (Figure 4A). Other haplotypes are represented by six or fewer individuals. Two of the haplotypes were present in all four regions, XXIII and VI, with five other haplotypes present in more than one region (XVI, IX, XXIV, XXVII, and XXV). Although the presence of high‐frequency haplotypes in multiple regions suggests gene flow is occurring between regions and between islands and the mainland, the haplotypes are not evenly distributed between regions, so there are limitations to the gene flow. The *S. americanus* haplotypes also exhibit a starburst pattern from haplotype XXIII (Figure 4B), but this did not coincide with any site‐specific historical population expansion as per Tajima's *D* (*p* > 0.05).

Additionally, both ectoparasites possess a haplotype that appears unique to Newfoundland. Seventeen specimens of *S. americanus* shared a haplotype that was only present in Newfoundland and shared between the four roost sites in the region (Figure 4, haplotype V). Six specimens of *M. insignis* collected at Salmonier Nature Park, Newfoundland, shared a haplotype only present at this site (Figure 3, haplotype V). The presence of a haplotype solely present in Newfoundland may suggest that the movement is primarily in the direction of Nova Scotia to Newfoundland or may represent the beginning of population expansion in Newfoundland.

**FIGURE 5:**
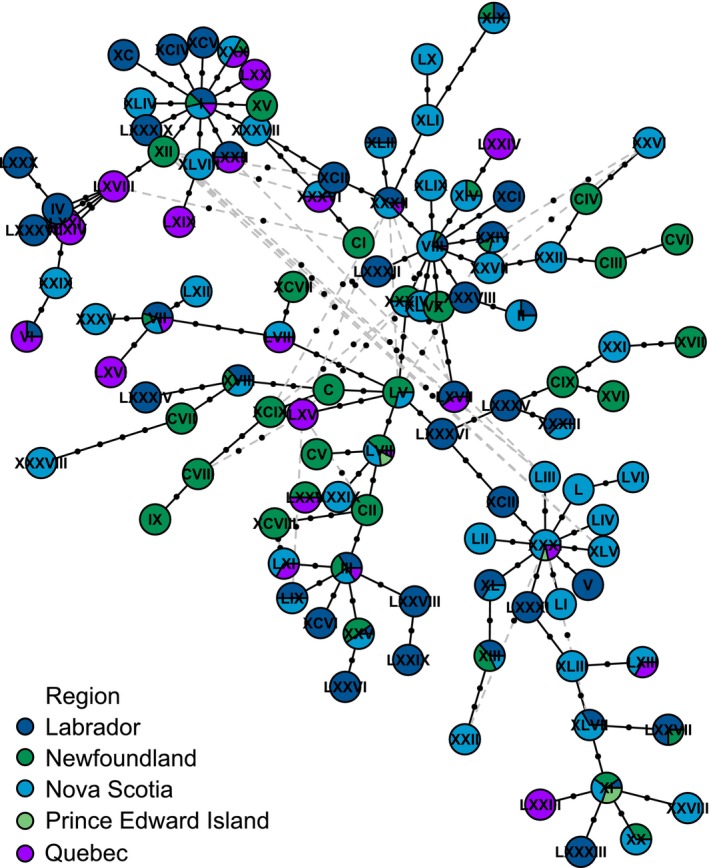
Visual representation of genetic relationships among *Myotis lucifugus* individuals and geographic regions inferred using a random minimized spanning tree algorithm. HVII sequence data from McLeod et al. (2015). Haplotypes are color‐coded according to collection region and Roman numerals correspond to haplotype numbers. Circle sizes are standardized to allow for the visualization of the starburst patterns. Black dots on lines represent missing mutational steps between haplotypes with solid black lines representing primary links between haplotypes and gray dashed lines representing alternative links between haplotypes. Networks were calculated and visualized using *pegas* in RStudio (Paradis 2010, R Core Team 2023).

Of the 625 *M. lucifugus* sequenced from McLeod et al. (2015), 54% were assigned to one of the five most abundant haplotypes, with 17% assigned to the most abundant haplotype (I). Of the 109 haplotypes assigned, 49 were singletons represented by only one sequence and 33 of the haplotypes are shared between regions. The *M. lucifugus* haplotypes exhibit starburst patterns around haplotypes I, III, VIII, and XXXI, four of the most abundant haplotypes (Figure 5). The starburst cluster around XXXI consists mostly of haplotypes sampled in Nova Scotia and Labrador, whereas the clusters around I, III, and VIII include more haplotypes sampled in Newfoundland and Quebec. These starbursts indicate the potential for historical population expansion in the little brown bat in this area of eastern Canada; however, the tests of neutrality were non‐significant for the overall population (*D* = −0.934, *p* > 0.10; D* = −1.091, *p* > 0.10; F* = −1.226, *p* > 0.10). Tajima's *D* was not significant at any individual sites.

## 4 Discussion

We identified varying levels of mtDNA genetic diversity in *M. insignis* and *S. americanus* in Atlantic Canada and low levels of genetic structure for both ectoparasites. As expected with the different life cycles of the two ectoparasite species, genetic diversity differed between them, with *M. insignis* exhibiting greater genetic diversity than *S. americanus* in terms of haplotype number, the number of variable sites, and the average number of nucleotide differences. The greater potential reproductive output of the fleas likely allows more opportunity for mutations to occur and build up in the species, translating to greater genetic diversity.

Our AMOVA and haplotype network results suggest gene flow occurs for *S. americanus* between roost sites; however, regional differentiation (*F*_ST_) is apparent between Newfoundland and the mainland. While there is less evidence of regional differentiation in *M. insignis*, there is a weak association between geographic distance and sequence divergence as well as evidence of population expansion in Labrador (Tajima's *D*, Fu and Li's F* and D*). The genetic structure of both ectoparasites is reflective of the medium to low genetic structure previously found in their host *M. lucifugus* (Burns et al. 2014; Johnson et al. 2015; McLeod et al. 2015) and was expected for *S. americanus* given its dependency on its bat hosts. Our hypothesis that *M. insignis* would exhibit greater genetic structure because of its roost‐dwelling life stages was not supported. Although neither species exhibited more than low levels of genetic structure between regions based on the AMOVA results, the greater number of *S. americanus* haplotypes across multiple regions suggests that the mites experience more dispersal with their bat hosts.

Parasites can act as biological tags for their host (Bruyndonckx et al. 2010; MacKenzie and Abaunza 2013; Catalano et al. 2014) and investigating the genetic structure of *M. insignis* and *S. americanus* has provided important insights into the movement ecology of the little brown bat in Atlantic Canada. Previous work by McLeod et al. (2015) on *M. lucifugus* found evidence of regional differentiation in eastern Canada between Newfoundland and the mainland. The regional differentiation between Newfoundland and the mainland for *S. americanus* is consistent with these findings. This is particularly prevalent for bats roosting at Salmonier Nature Park, Newfoundland, which showed high regional differentiation when compared to roost sites in Labrador (*F*_ST_ = 0.587, *p* < 0.001) and low to medium levels of differentiation from most roost sites in Nova Scotia (0.299 ≤ *F*_ST_ ≤ 0.455, *p* < 0.001). Gene flow appears to be occurring between bats at Pynn's Brook, Newfoundland, and bats in Nova Scotia, allowing for transmission of *S. americanus* and gene flow between the mite populations. There are still low to medium levels of differentiation between mites at Pynn's Brook and most roost sites in Labrador (0.263 ≤ *F*_ST_ ≤ 0.438, *p* < 0.001). The connectivity of bats and their mites between Newfoundland and Labrador may be restricted due to yet undetermined geographic, climatic, or behavioral barriers that are not present or are less restrictive between Nova Scotia and the western portion of Newfoundland (McLeod et al. 2015).

Host‐specific parasites and permanent parasites are expected to show higher levels of genetic structure as their dispersal and survival are tightly linked to their host (Nadler 1995). Despite being generally considered host‐specific, the genetic structure of spinturnid mites appears to be strongly dependent on the movement ecology and behavior of their host species (Bruyndonckx, Henry, et al. 2009; Zamora‐Mejías et al. 2022). The spread of haplotypes across regions for *S. americanus* is consistent with gene flow or transmission of mites between roost sites and regions within Atlantic Canada. Although *S. americanus* populations are likely greatest during the spring and summer (Orlova et al. 2018), the mites have been identified from *M. lucifugus* and *Myotis septentrionalis* at swarming sites in Nova Scotia and New Brunswick (Poissant and Broders 2008; Czenze and Broders 2011). Swarming locations may provide opportunities for the transfer of mites between bats from different summer roosts and potentially between different *Myotis* species. At least some female *M. lucifugus* are philopatric, where they return to the roosts where they were born (Dixon 2011), or show some combination of roost fidelity and social preference leading to the return of female bats to the same roosts year over year (Sunga et al. 2024). This consistent return of female bats to maternity roosts provides a reliable resource for ectoparasites, whereas the dispersal of other females and male bats provides an avenue for ectoparasite transmission to other areas (Dixon 2011).

The fleas do not show as strong a regional differentiation between Newfoundland and the mainland as the mites; however, they do show evidence of structure and population growth. There appear to be two geographic clusters for haplotypes within Labrador and Nova Scotia, with some overlap between these two regions and with Newfoundland. Two roost sites in Labrador exhibited medium levels of regional differentiation with roost sites in Nova Scotia (0.314 ≤ *F*_ST_ ≤ 0.556, *p* < 0.001) which may, in part, be accounted for by isolation by distance (Mantel test *r* = 0.105, *p* = 0.01). Although *M. insignis* adults are present at swarming sites (Czenze and Broders 2011), reproduction of these fleas occurs during the summer, and the pupa is proposed as the overwintering life stage (Smith and Clay 1988). Adult fleas dispersing to swarming sites and hibernacula with their hosts may have limited effects on gene flow within this flea species, contributing to regional differentiation. The population expansion of *M. insignis* in Labrador is particularly apparent, as represented by the starburst pattern around haplotype XIII and the Tajima's *D* results (Charlottetown Junction: *D* = −1.658, *p* = 0.01; Anne Marie Lake: *D* = −2.129, *p* = 0.00). This expansion is likely a result of *M. lucifugus* being restricted to a limited number of anthropogenic structures suitable for roosting. Ectoparasite infestations may be energetically costly for bats (Giorgi et al. 2001) and bats have been shown to change roosts to avoid ectoparasite infections (Bartonička 2008). The lack of alternative roost options in Labrador prevents the bats from switching roosts to avoid ectoparasites, allowing the fleas to persist at larger population sizes. The differences in life history and reproductive strategy between *M. insignis* and *S. americanus* likely mean that the fleas are better suited to exploit this stable host supply.

Both ectoparasites likely underwent a population expansion in eastern Canada following the arrival of their host in the area after the Pleistocene deglaciation (Burns et al. 2014). All three species exhibit starburst patterns in their haplotype networks, suggesting historical population expansion. The tests of neutrality for all sampled *S. americanus* and *M. insignis* also support the hypothesis that these species exhibited past population expansion. The local population growth of *M. insignis* in Labrador was likely more recent, following the continued northward expansion of the range for *M. lucifugus* . The presence of medium abundance haplotypes in Newfoundland only for both ectoparasites may indicate the potential for population growth at those roost sites during the time of collection.

*Myotis lucifugus* and other hibernating bats in North America have experienced drastic declines in population numbers following the spread of the white‐nose syndrome‐causing fungus, *Pseudogymnoascus destructans* (Lorch et al. 2016; Environment Canada 2018). Populations of *M. insignis* and *S. americanus* are likely experiencing pressures related to declines in *M. lucifugus* and other host species, and there is the potential for significant genetic restructuring of these two ectoparasite species. The secondary effects of this disease on the bat ectoparasites are currently unknown, but local extirpations and genetic bottlenecks are possible. Both *S. americanus* and *M. insignis* have previously been recorded from the big brown bat ( *Eptesicus fuscus* ; Phillips 1966; Dick et al. 2003) which appears to be less susceptible to white‐nose syndrome (Pearce and O'Shea 2007; Frank et al. 2014). If *E. fuscus* is a viable alternative host for *S. americanus* and *M. insignis* , it may act as a reservoir for these species. The survival of these ectoparasites is dependent on the long‐term viability of their *Myotis* spp. hosts and the potential for reservoir populations on alternative hosts like *E. fuscus*.

## 5 Conclusion

The results of our study highlight the importance of considering host–parasite dynamics and parasite life history when investigating the genetic structure of parasites. The differences in host–parasite dynamics between *M. insignis* and *S. americanus* and their host likely contributed to their varying levels of genetic diversity, while the movement ecology of *M. lucifugus* maintained gene flow between populations. This research highlights the importance of host mobility and sociality in the maintenance of gene flow between parasite populations at large scales. If this pattern of mirroring of host genetic structure is consistent for other threatened or endangered host species, parasites may provide additional insight into the behaviors and movements of the hosts that can help inform conservation and management decisions. Additionally, understanding how aspects of both parasite and host biology influence the host–parasite relationship at multiple scales is necessary for being able to predict how these dynamics may be affected by disturbances such as climate change, disease, and species introductions, and land use changes.

## Author Contributions

**Alexandra H. Sauk:** conceptualization (equal), data curation (equal), formal analysis (lead), investigation (lead), methodology (equal), validation (lead), visualization (lead), writing – original draft (lead), writing – review and editing (equal). **Hugh G. Broders:** conceptualization (equal), data curation (equal), formal analysis (supporting), funding acquisition (lead), investigation (equal), methodology (supporting), project administration (lead), resources (lead), supervision (lead), validation (supporting), visualization (supporting), writing – original draft (supporting), writing – review and editing (equal).

## Disclosure

Benefit generated: this research provided more insight into the relationship between *Myotis lucifugus* and its most common ectoparasites, which can inform management decisions regarding roost availability for the recovery of *Myotis lucifugus* in Atlantic Canada and elsewhere.

## Conflicts of Interest

The authors declare no conflicts of interest.

## Supporting information


Appendix S1.

## Data Availability

COI sequences and metadata for *Spinturnix americanus* and *Myodopsylla insignis* specimens in this paper will be publicly available on BOLD after publication. Specimen sequences are available on NCBI GenBank under accessions PQ764161 – PQ764470. Haplotypes from MacLeod et al. are available in NCBI GenBank under PopSet: 820715873. Benefit generated: COI sequences of *Spinturnix americanus* and *Myodopsylla insignis* contribute to the BOLD mission of barcoding all species on the planet and provide some of the first publicly accessible sequences for identified specimens of these species with associated photographs and metadata.
